# Supplementary material for: Maintenance of intestinal CX3CR1+ macrophage homeostasis defines post-treatment control in SIV-infected macaques
Source: Nat Commun. 2026 Feb 24;17:3111. doi: 10.1038/s41467-026-69848-5 (PMC13039934; doi:10.1038/s41467-026-69848-5)
Supplement: Supplementary file 1 — Supplementary Information [file 41467_2026_69848_MOESM1_ESM.pdf]

Supplementary Information for

Maintenance of intestinal CX3CR1^{+} macrophage homeostasis defines post-treatment control in SIV-infected macaques

Stéphane Hua et al.

The PDF file includes:

Supplementary Tables S1-S4

Supplementary Figures 1-12

Table S1. Characteristics of the animals included in the study

Group	ID	Sex	MHC Haplotype	Age at inclusion (year)	Weight at inclusion (kg)	Days post-infection at euthanasia	Days post-ATI at euthanasia
SIV-	1LU8	F	n.a.	7	6.72	-	-
SIV-	1MU8	F	n.a.	7	4.51	-	-
SIV-	CA147	F	n.a.	5	4.12	-	-
SIV-	CA577D	F	M3/M2	4	3.83	-	-
SIV-	CA886	F	n.a.	4	4.15	-	-
SIV-	CBD014	M	M3M1/M3M1	3	4.2	-	-
SIV-	BA1R	F	M7/M1	4	3.77	-	-
SIV-	BT364	F	M1/M5	6	4.38	-	-
SIV-	CB136	F	n.a.	5	4.5	-	-
SIV-	CB993	F	M2/M2M5	5	4.47	-	-
SIV-	CB996	F	M3/M1M3	5	4.32	-	-
SIV-	CGD003	F	M5/M6	4	4.04	-	-
SIV+	CDJ017	M	M1/M2M5	5	5.51	335	-
SIV+	CDJ031	M	M2/M3	5	7.67	342	-
SIV+	CDJ036	M	M4/M1M4	5	7.17	177	-
SIV+	CDJ052	M	M2/M1M5	5	7.25	356	-
SIV+	CDJ053	M	M1/M1M2	5	7.69	349	-
SIV+	CDK079	M	M2/M5	5	5.71	363	-
SIV+	BA881J	M	M3/M1	8	12.12	462	-
SIV+	BA989J	M	M4/M1	6	12.3	358	-
SIV+	BB750G	M	M3/M2M4	7	10.95	338	-
SIV+	BC618D	M	M4M3/M2	7	13.21	331	-
SIV+	BC803C	M	M4/M2	8	8.8	336	-
SIV+	CB762B	M	M1M5M3/M3	6	10.25	462	-
PTC	BA736J	M	M1M2M1/M4	5	5.5	1157	394
PTC	BA922I	M	M3/M1	6	5.4	1143	380
PTC	BB123J	M	M2M1/M3	5	5.8	1147	384
PTC	CA706F	M	M5/M4M1M2	4	4.4	1154	391
PTC	BA797I	M	M1/M3M4	7	7.65	1112	378
PTC	BB9I	M	M1/M2M5	7	9.05	1115	381
PTC	BB799G	M	M3M5/M2M3	4	6.5	1108	374
PTC	CB806C	M	M1M5M3/M2M3	5	6.54	1119	385
PTC	BA979I	M	M4/M2	7	8.55	1126	230
PTC	CCB090	M	M3M1M3/M5	5	5.2	1133	237
NC	BA777K	M	M2/M1M5	5	6.7	1010	247
NC	BA733K	M	M1M4/M2	7	9.67	1140	244
NC	BB340E	M	M3M1/M4	7	8.5	1122	226

Table S2. Panels for lymphocyte phenotyping

A) Lymphocyte Activation and Trafficking Panels

Antibody	Fluorochrome	Clone	Reference	Supplier	Panel
Fixable viability stain	Blue for UV excitation	-	L23105	Invitrogen	P1 & 2
CD45	PerCp-Vio700	REA1023	130-117-197	Miltenyi Biotec	P1 & 2
CD3	BUV395	SP34-2	564117	BD Horizon	P1 & 2
CD4	Viobright 515	REA623	130-114-535	Miltenyi Biotec	P1 & 2
CD8	Alexa-Fluor 700	RPA-T8	2105140	SONY	P1 & 2
HLA-DR	APC-Vio770	REA805	130-111-792	Miltenyi Biotec	P1 & 2
CD28	BV650	CD28.2	302946	Biolegend	P1
CD95	BV510	DX2	2128200	SONY	P1
CCR7	PE	REA546	130-119-583	Miltenyi Biotec	P1
CD45RA	Vioblue	T6D11	130-113-360	Miltenyi Biotec	P1
CD25	APC	REA945	130-115-535	Miltenyi Biotec	P1
CCR5	BV786	3A9	565001	BD Horizon	P1
CD69	PE-Vio615	REA824	130-112-617	Miltenyi Biotec	P1
KI67	PE-Cy7	B56	561283	BD Pharmingen	P1
α4β7	Alexa Fluor 647	Hu117	FAB10078R	R&D Systems	P2
CD62L	BV786	SK11	565311	BD Horizon	P2
CCR9	PE-Dazzle 594	L053E8	358918	Biolegend	P2
PD1	PE-Cy7	EH12.1	561272	BD Pharmingen	P2

B) Regulatory T Cell Panel

Antibody	Fluorochrome	Clone	Reference	Supplier	Panel
Fixable viability stain	Blue for UV excitation	-	L23105	Invitrogen	P3
CD45	PerCp-Vio700	REA1023	130-117-197	Miltenyi Biotec	P3
CD3	BUV395	SP34-2	564117	BD Horizon	P3
CD4	Viobright 515	REA623	130-114-535	Miltenyi Biotec	P3
CD8	Alexa-Fluor 700	RPA-T8	2105140	SONY	P3
HLA-DR	APC-Vio770	REA805	130-111-792	Miltenyi Biotec	P3
CD28	BV650	CD28.2	302946	Biolegend	P3
CD95	BV510	DX2	2128200	SONY	P3
CCR7	PE	REA546	130-119-583	Miltenyi Biotec	P3
CD45RA	Vioblue	T6D11	130-113-360	Miltenyi Biotec	P3
CD25	APC	REA945	130-115-535	Miltenyi Biotec	P3

CCR5	BV786	3A9	565001	BD Horizon	P3
FOXP3	PE-Dazzle 594	206D	320126	Biolegend	P3
CD127	PE-Vio770	MB15-18C9	130-113-412	Miltenyi	P3

C) Cytokine Profile Panel

Antibody	Fluorochrome	Clone	Reference	Supplier	Panel
Fixable viability stain	Blue for UV excitation	-	L23105	Invitrogen	P4
CD45	PerCp-Vio700	REA1023	130-117-197	Miltenyi Biotec	P4
CD3	V500	SP34-2	560770	BD	P4
CD8	BV650	RPA-T8	563821	BD Horizon	P4
CD4	BV421	L200	562842	BD Horizon	P4
IL-22	APC	IL22JOP	17-7222-82	eBioscience	P4
IFN-g	APC-H7	B27	3132620	SONY	P4
IL-17A	PE-Vio615	REA1063	130-118-247	Miltenyi Biotec	P4
IL-4	PE-Vio770	REA895	130-114-844	Miltenyi Biotec	P4

Table S3. Panels for myeloid cells phenotyping

A) Core Myeloid Panel

Antibody	Fluorochrome	Clone	Reference	Supplier	Panel
Fixable viability stain	Blue for UV excitation	-	L23105	Invitrogen	P1
CD45	PerCp	D058-1283	558411	BD Pharmingen	P1
CD3	V500	SP34-2	560770	BD Horizon	P1
CD8	BV650	RPA-T8	563821	BD Horizon	P1
CD14	Alexa-Fluor 700	M5E2	557923	BD Pharmingen	P1
CD16	PE-CF594	3G8	562293	BD Horizon	P1
CD20	BV711	2H7	563126	BD Horizon	P1
HLA-DR	APC-Cy7	G46-6	561358	BD Pharmingen	P1
CD11c	APC	S-HCL-3	333144	BD Pharmingen	P1
CD123	PE-Cy7	7G3	560826	BD Pharmingen	P1
CD64	V450	10,1	561202	BD Horizon	P1
CD103	PE	Bly7	12-1038	eBioscience	P1
CX3CR1	FITC	2A9-1	341605	Biolegend	P1

B) Myeloid Functional Panels

Antibody	Fluorochrome	Clone	Reference	Supplier	Panel
Fixable viability stain	Blue for UV excitation	-	L23105	Invitrogen	P 2,3,4
CD45	PerCp	D058-1283	558411	BD Pharmingen	P 2,3,4
CD3	BV650	SP34-2	563916	BD Horizon	P 2,3,4
CD8	BV650	RPA-T8	563821	BD Horizon	P 2,3,4
CD14	Alexa-Fluor 700	M5E2	557923	BD Pharmingen	P 2,3,4
CD16	BUV395	3G8	563785	BD Horizon	P 2,3,4
CD20	BV650	2H7	563780	BD Horizon	P 2,3,4
HLA-DR	APC-Cy7	G46-6	561358	BD Pharmingen	P 2,3,4
CD11c	BV421	3,9	2108140	SONY	P 2,3,4
CD123	PE-Cy7	7G3	560826	BD Pharmingen	P 2,3,4
CD64	Viogreen	REA978	130-116-203	Miltenyi Biotec	P 2,3,4
CD103	PE	Bly7	12-1038	eBioscience	P 2,3,4
CX3CR1	FITC	2A9-1	341605	Biolegend	P 2,3,4
CD40	PE-Dazzle 594	5C3	334342	Biolegend	P2
CD83	APC	REA714	130-110-504	Miltenyi Biotec	P2

CCR5	BV786	G46-17	565001	BD Horizon	P2
CD69	PE-Vio615	REA824	130-112-617	Miltenyi Biotec	P3
CD86	APC	REA968	130-116-161	Miltenyi Biotec	P3
CD80	BV786	L307.4	564159	BD Horizon	P3
CCR9	PE-Dazzle 594	L053E8	358918	Biolegend	P4
α4β7	Alexa-Fluor 647	Hu117	FAB10078R	R&D Systems	P4
CD62L	BV786	SK11	565311	BD Horizon	P4

Table S4. Panel for neutrophil phenotyping

Antibody	Fluorochrome	Clone	Reference	Supplier	Panel
Fixable viability stain	Blue for UV excitation	-	L23105	Invitrogen	Neutrophil
CD64	BUV737	10.1	564425	BD Horizon	Neutrophil
CD14	VioBlue	REA599	130-110-524	Miltenyi Biotec	Neutrophil
CD45	VioGreen	REA1023	130-117-193	Miltenyi Biotec	Neutrophil
CD3	BV650	SP34.2	563916	BD Horizon	Neutrophil
CD123	BV650	7G3	563405	BD Horizon	Neutrophil
CD8 a	BV650	RPAT8	563821	BD Horizon	Neutrophil
CD20	BV650	2H7	563780	BD Horizon	Neutrophil
CD62L	BV711	SK11	565040	BD Horizon	Neutrophil
CD11b	FITC	REA713	130-110-552	Miltenyi Biotec	Neutrophil
CD10	PerCP-Cy5.5	Hl10a	312216	BioLegends	Neutrophil
CD125	PE	REA705	130-110-544	Miltenyi	Neutrophil
PD-L1	PE-Dazzle594	29E.2A3	329732	BioLegends	Neutrophil
CD101	PE-Vio770	REA954	130-115-832	Miltenyi Biotec	Neutrophil
CD32a	AF647	IV.3	60012	Stemcell	Neutrophil
HLA-DR	AF700	L234	307626	BioLegends	Neutrophil
CD66abce	APC-Vio770	TET2	130-119-847	Miltenyi Biotec	Neutrophil

Supplementary figure 1

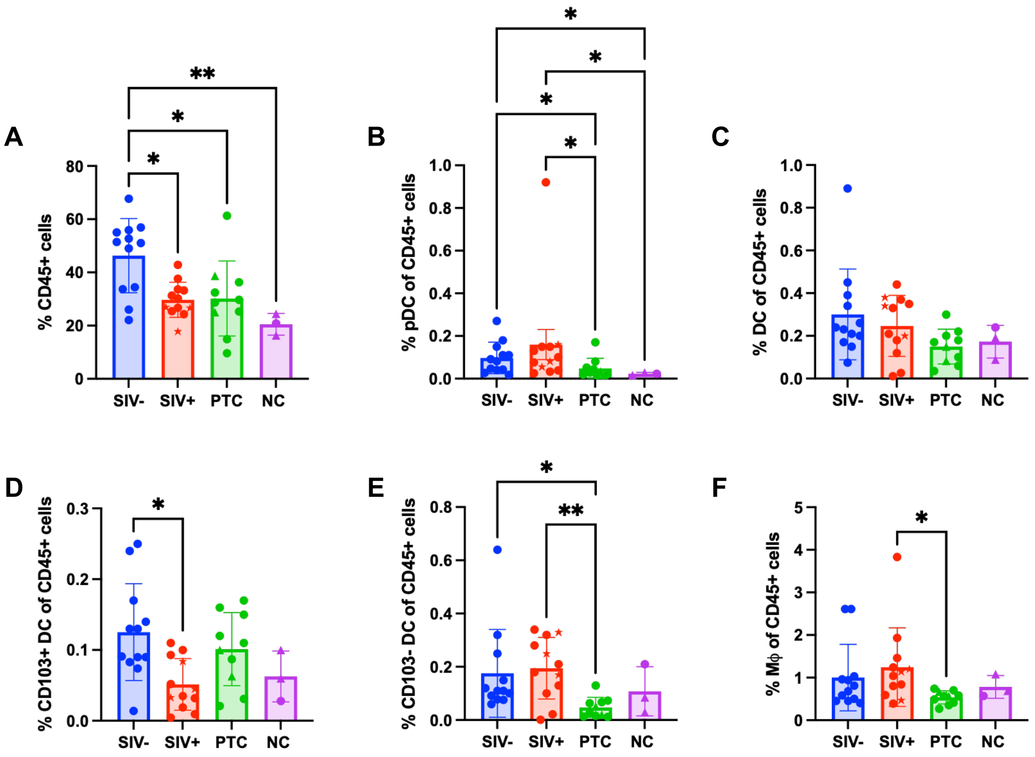

Supplementary Figure 1. Innate myeloid cell composition in the sigmoid colon of SIV-infected and control animals. (**A–F**) Frequencies of innate immune cell populations among $CD45^+$ cells in the sigmoid colon across SIV- (n=12), SIV+ (n=12), PTC (n=10), and NC (n=3) animals: total $CD45^+$ cells (A), plasmacytoid dendritic cells (pDC; B), conventional dendritic cells (DC; C), $CD103^+$ DCs (D), $CD103^-$ DCs (E), and macrophages (F). *Kruskal–Wallis with Benjamini–Krieger–Yekutieli (FDR) correction*; $q < 0.05$ (*), $q < 0.01$ (**), $q < 0.001$ (***). In the histograms, symbol shapes indicate the study group: circles represent SIV^- and SIV^+ samples as well as PTCs and NCs from Group 3; stars represent natural controllers; triangles represent PTCs and NCs from Group 4. Symbol shapes and colour coding are consistent across all figures. Colours denote experimental groups: blue, SIV^-; red, SIV^+; green, PTC; magenta, NC. Source data are provided as a Source Data file.

Supplementary figure 2

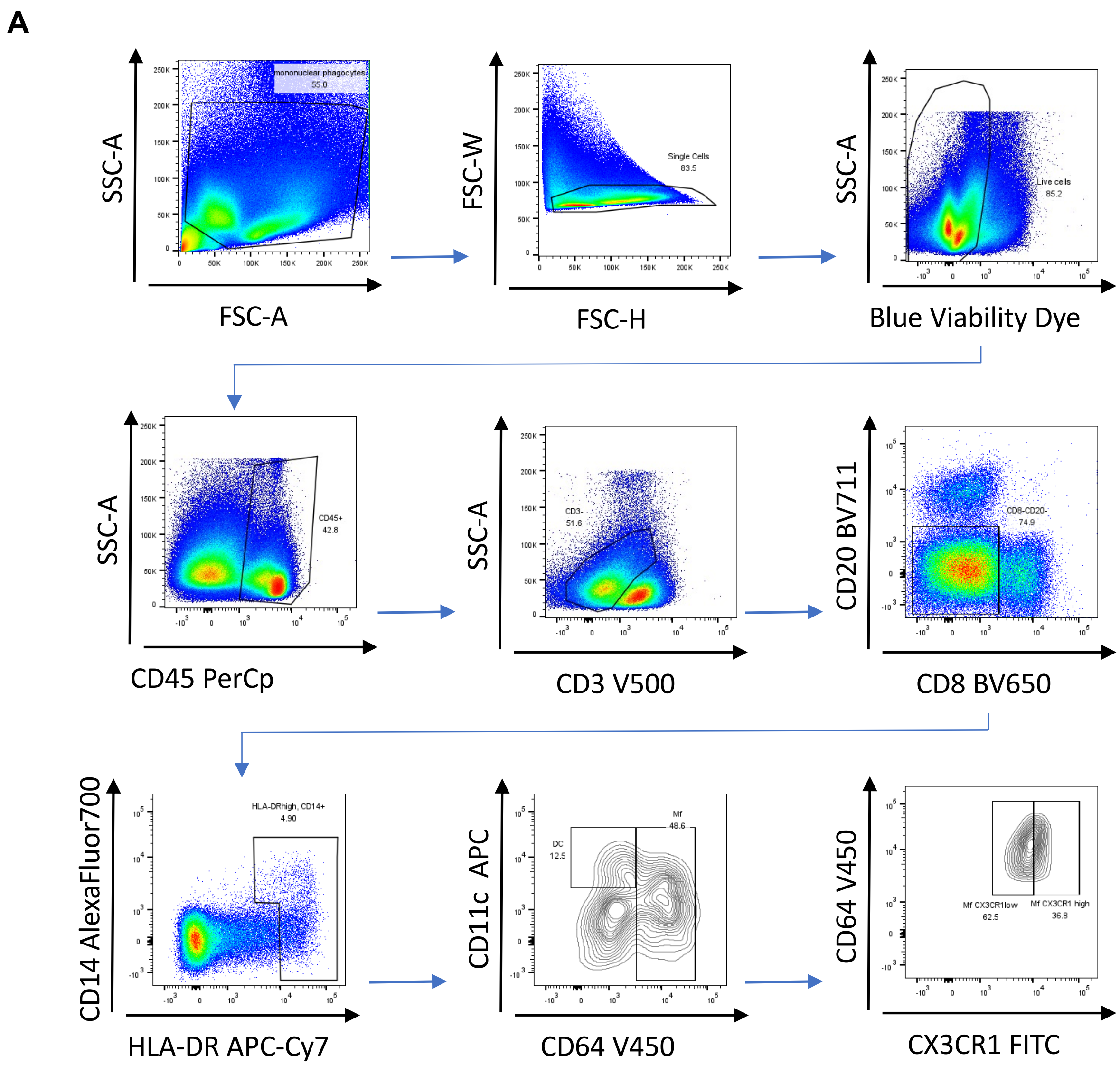

Supplementary figure 2

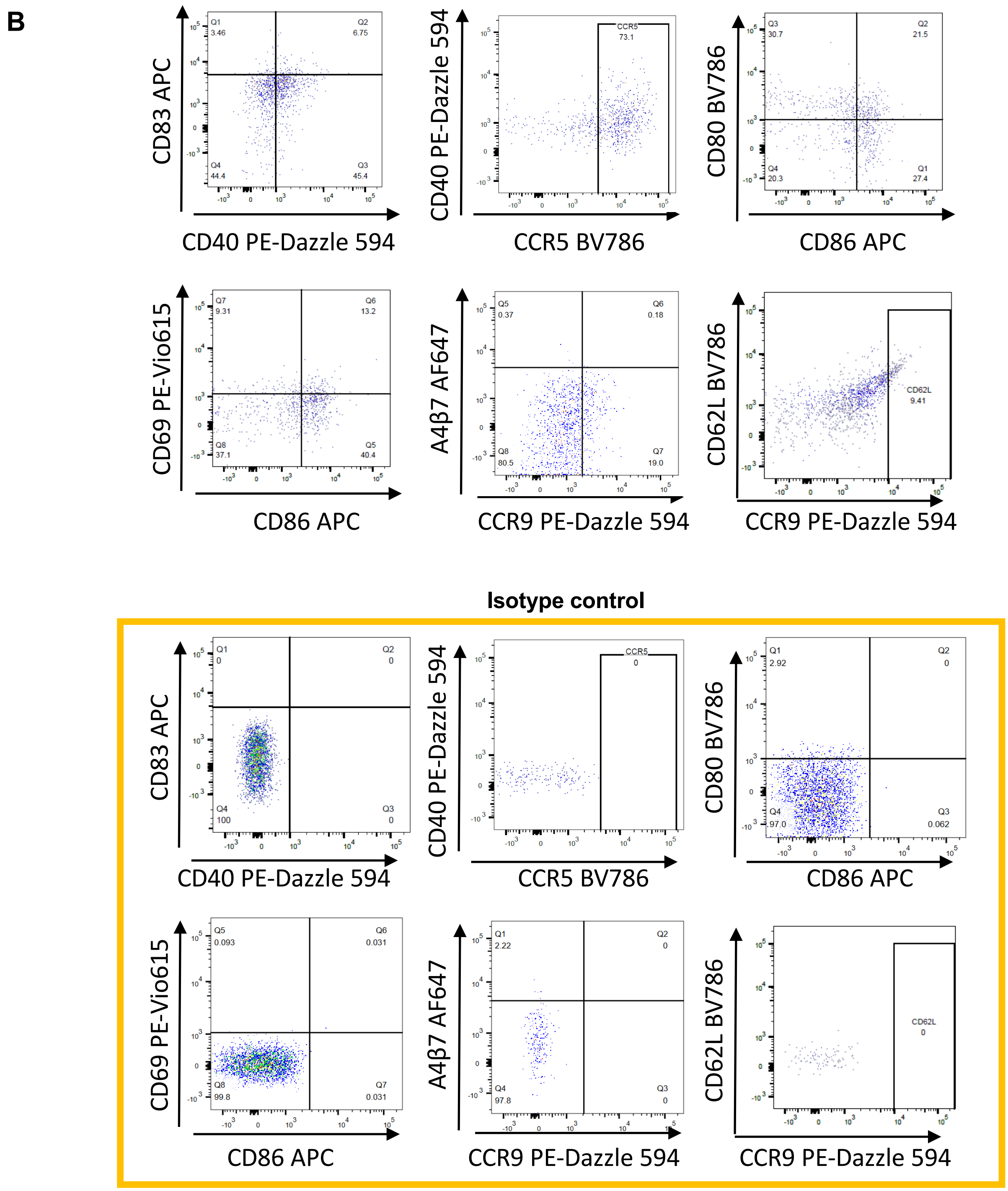

Supplementary Figure 2. Flow cytometry strategy for phenotypic analysis of intestinal macrophages. (**A**) Representative gating strategy used to identify macrophages in the sigmoid colon. Single cells are identified by plotting FSC-A against FSC-H. Mononuclear cells are identified by size by plotting FSC-A against SSC-A, and live cells are defined as LIVE/DEAD Fixable Blue Dead Cell stain negative. Among CD45+ leukocytes, an initial gate on $CD3^{-}CD8^{-}$ $CD20^{-}$ cells allowed exclusion of T lymphocytes, NK and B cells, followed by a gate on HLA-$DR^{high}CD14^{+}$ cells. Macrophages were identified based on the expression of CD11c and CD64 and included $CD11c^{+}CD64^{+}$ and $CD11c^{-}CD64^{+}$ cells. DCs were defined as $CD11c^{+}CD64^{-}$cells. Expression of CX3CR1 by macrophages was further assessed. (**B**) Expression profiles of surface markers associated with macrophage activation, homing, and maturation: CD83, CD40, CCR5, CD80, CD86, CD69, CCR9, α4β7, and CD62L (upper panels). Corresponding isotype controls are shown in the lower panels (yellow box).

Supplementary figure 3

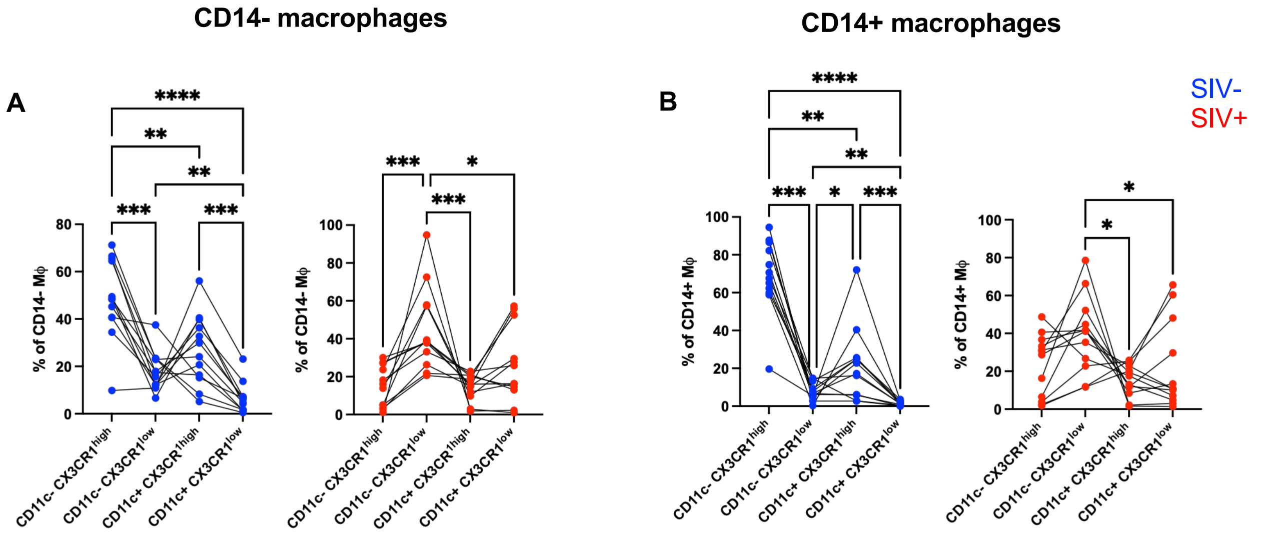

Supplementary Figure 3. Differential expression of CD11c and CX3CR1 in CD14⁻ and CD14⁺ intestinal macrophage subsets. (**A–B**) Expression of CD11c and CX3CR1 within CD14⁻ (A) and CD14⁺ (B) macrophage subsets in the sigmoid colon of SIV⁻ (n=12) and SIV⁺ (n=12) animals. *Wilcoxon matched-pairs signed-rank test (non-parametric); p* < 0.05 (***), *p < 0.01* (**), *p* < 0.001 (***). Source data are provided as a Source Data file.

Supplementary figure 4

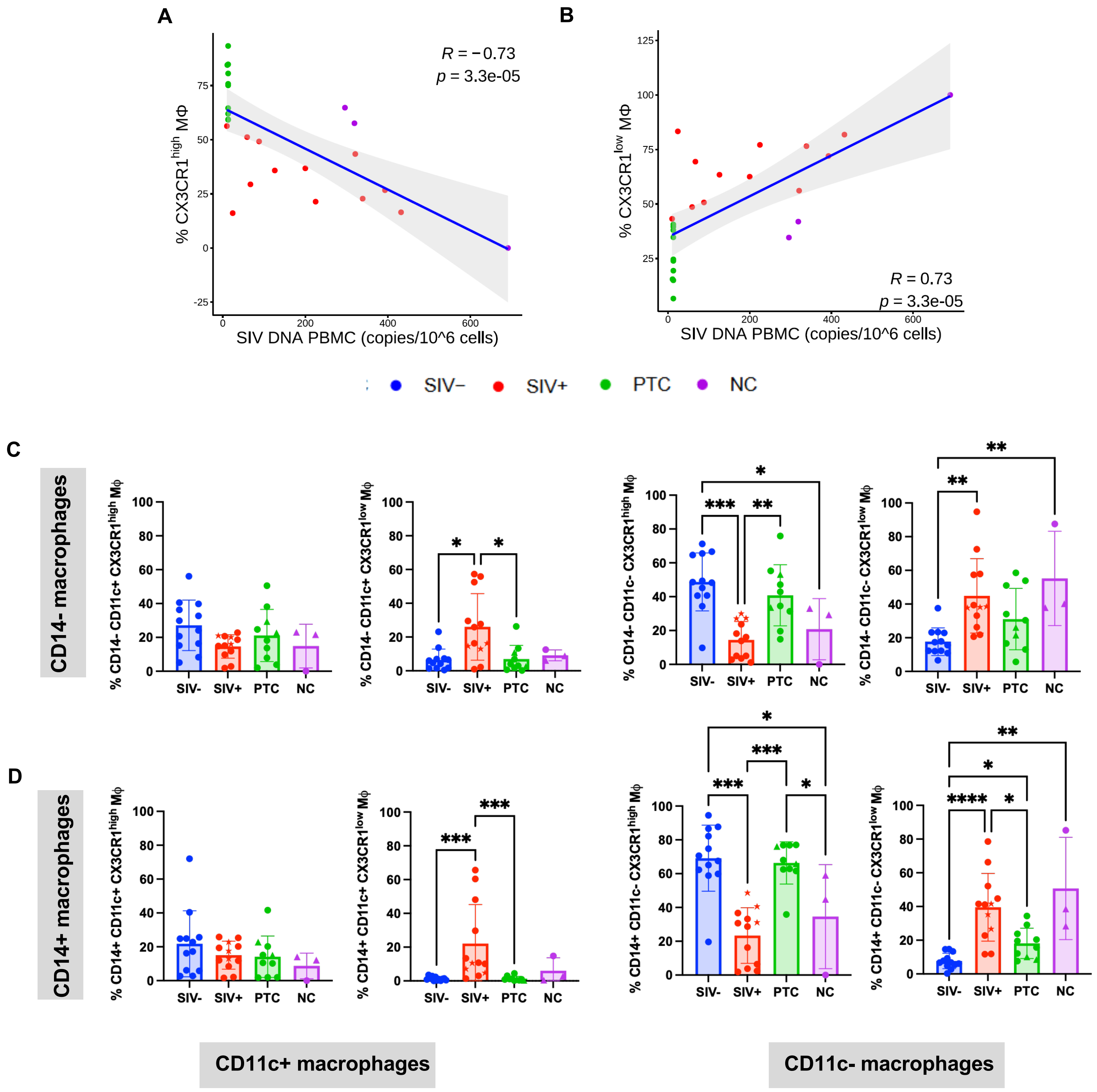

Supplementary Figure 4. Association of intestinal macrophage phenotypes with systemic viral burden. (**A–B**) Scatter plots show Spearman correlations between the frequency of $CX3CR1^{high}$ (A) or $CX3CR1^{low}$ (B) macrophages in the sigmoid colon and SIV DNA levels in PBMCS. Spearman correlation coefficients (R) and p-values are indicated in each panel. Blue lines depict linear regression fits with 95% confidence intervals (gray shading). Confidence intervals are centered around the mean of the x value. All Spearman correlation were two-sided. (**C–D**) Frequencies of $CD11c^{+}$ and $CD11c^{-}$ macrophages co-expressing CX3CR1 ^{high} or CX3CR1 ^{low} within $CD14^{-}$ (C) and $CD14^{+}$ (D) macrophage subsets across SIV- (n=12), SIV+ (n=12), PTC (n=10), and NC (n=3) animals. *Kruskal–Wallis with Benjamini–Krieger–Yekutieli (FDR) correction*; $q < 0.05$ (*), $q < 0.01$ (**), $q < 0.001$ (***). Source data are provided as a Source Data file.

Supplementary figure 5

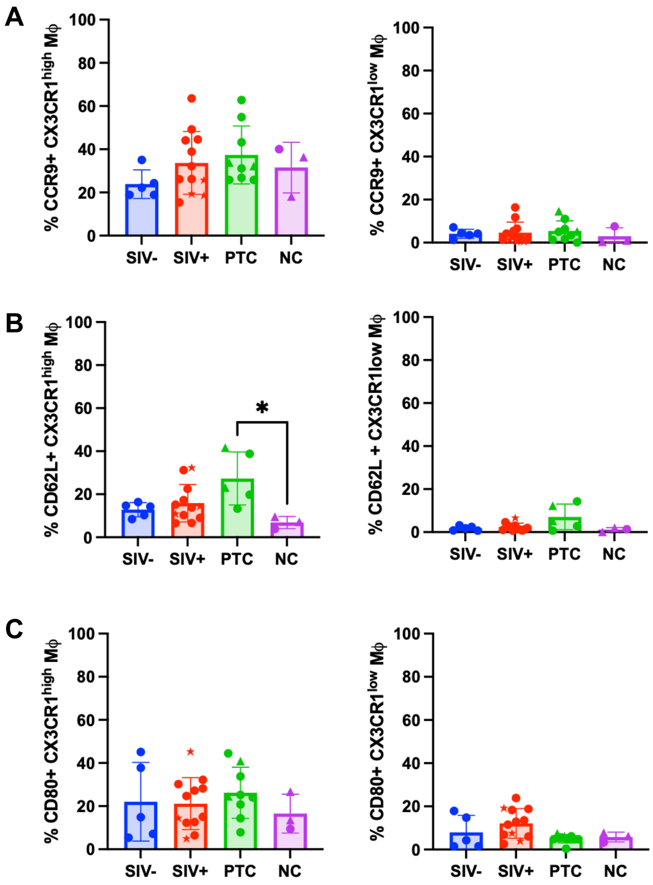

Supplementary Figure 5. Expression of CCR9, CD62L, and CD80 by CX3CR1-defined intestinal macrophage subsets. (**A–C**) Frequencies of CCR9^{+} (A), CD62L^{+} (B), and CD80^{+} (C) cells among CX3CR1^{high} and CX3CR1^{low} macrophages in the sigmoid colon of SIV- (n=5), SIV+ (n=12), PTC (n=9), and NC (n=3) animals. *Kruskal–Wallis with Benjamini–Krieger–Yekutieli (FDR) correction*; q < 0.05 (*), q < 0.01 (**), q < 0.001 (***). Source data are provided as a Source Data file.

Supplementary figure 6

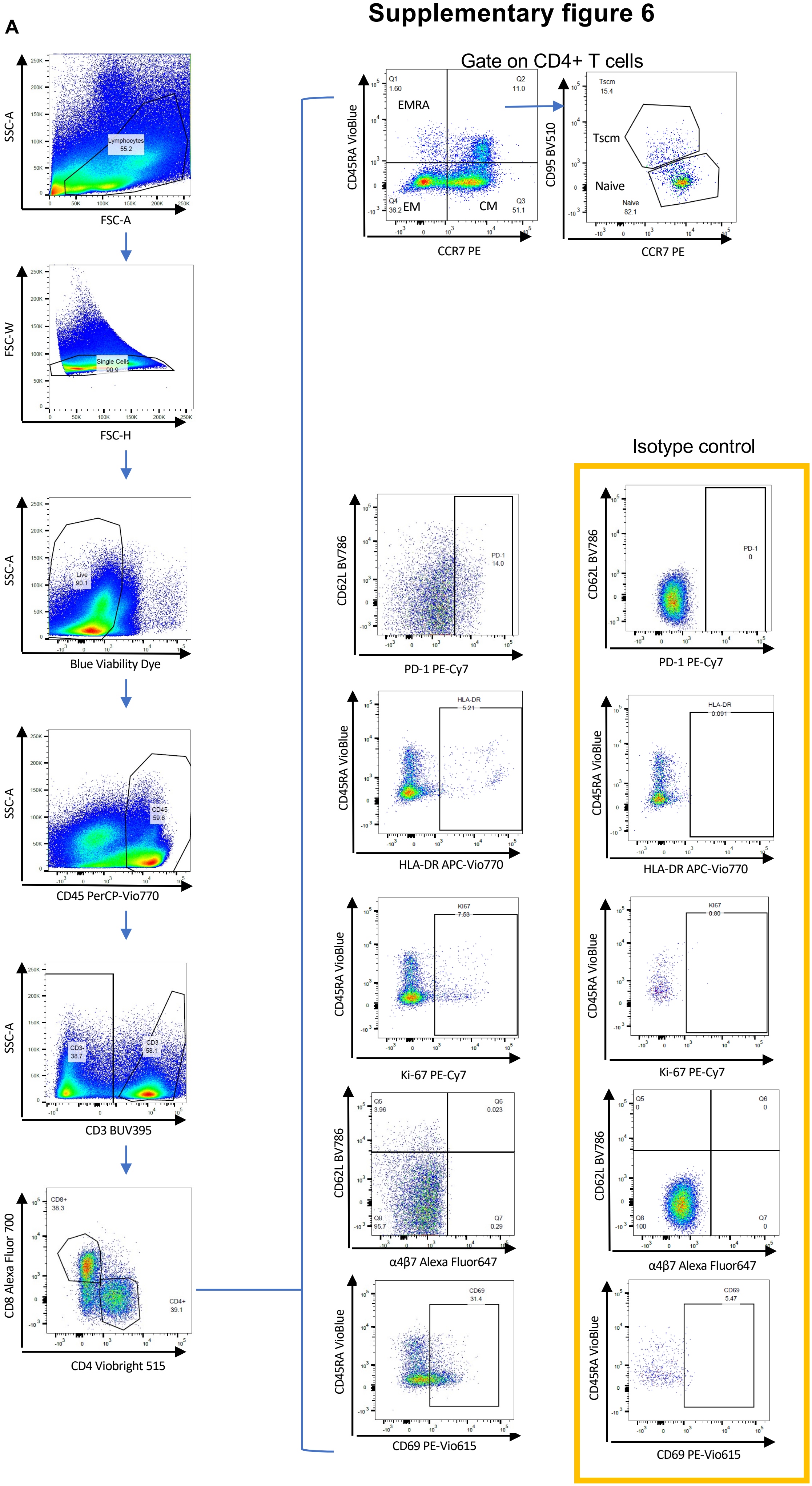

B

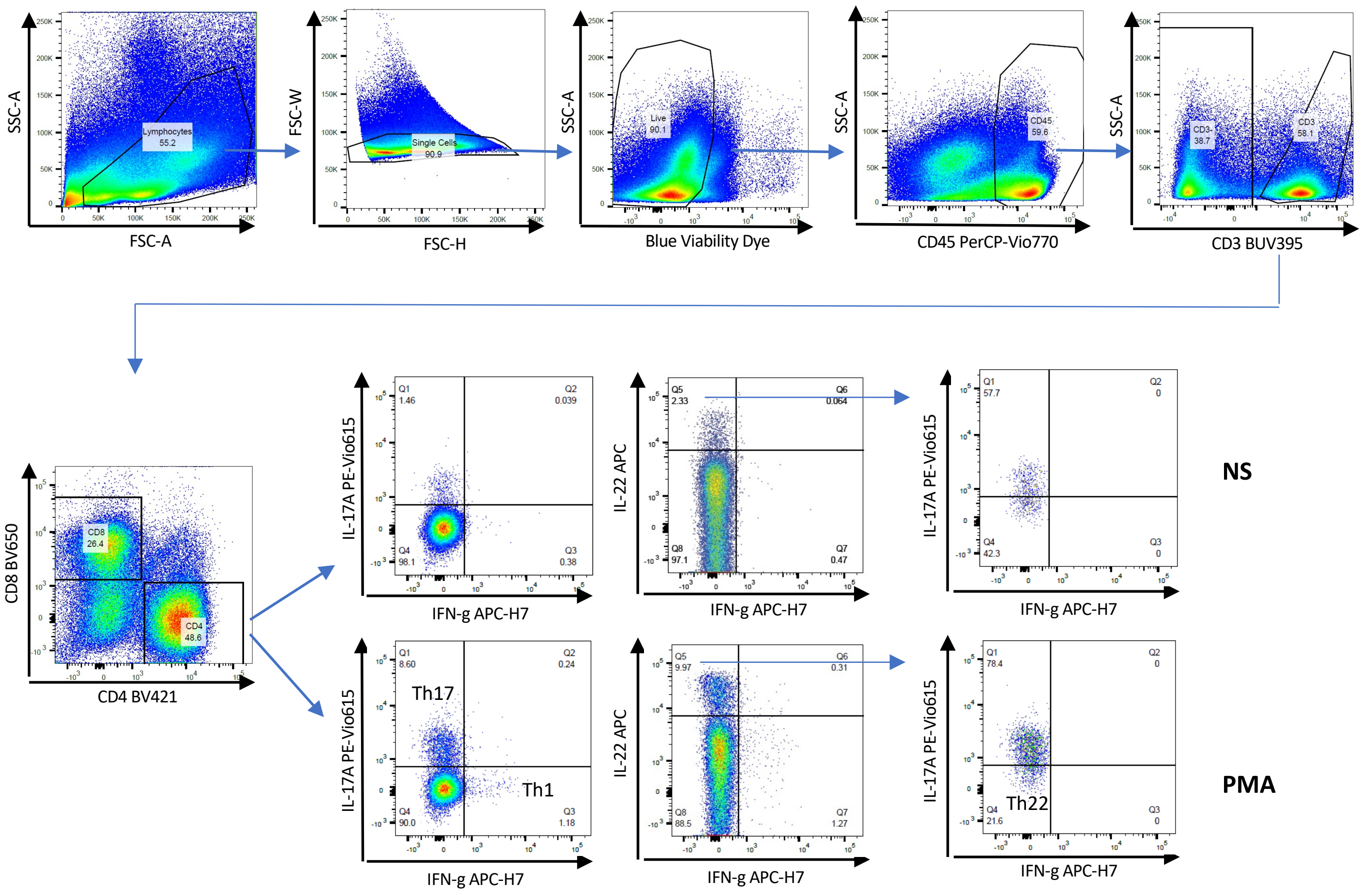
SSC-A
FSC-A
Lymphocytes 55.2
FSC-W
FSC-H
Single Cells 90.9
Blue Viability Dye
Live 90.1
CD45 PerCP-Vio770
CD45 59.6
CD3 BUV395
CD3- 38.7
CD3 58.1
CD8 BV650
CD4 BV421
CD8 26.4
CD4 48.6
IL-17A PE-Vio615
IL-22 APC
IFN-g APC-H7
NS
PMA
Th17
Th1
Th22

C

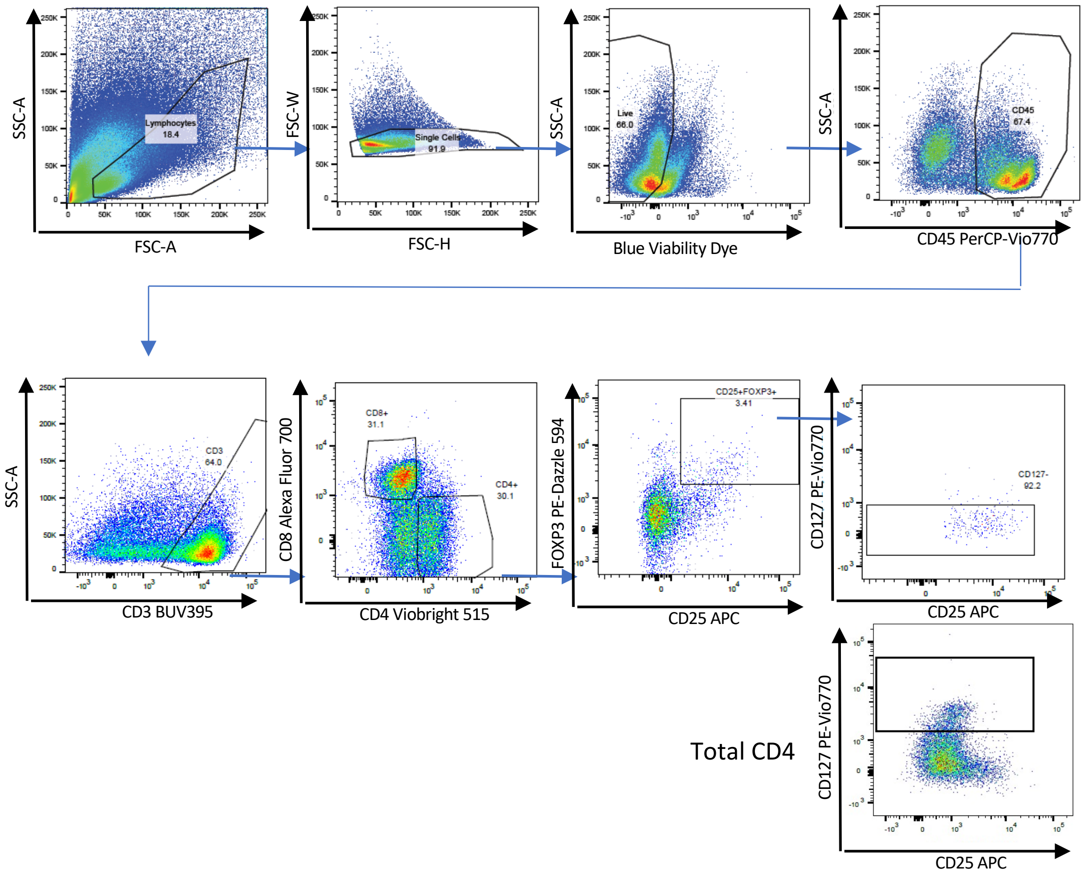
SSC-A
FSC-A
Lymphocytes 18.4
FSC-W
FSC-H
Single Cells 91.9
Blue Viability Dye
Live 66.0
CD45 PerCP-Vio770
CD45 67.4
CD3 BUV395
CD3 64.0
CD8 Alexa Fluor 700
CD4 Viobright 515
CD8+ 31.1
CD4+ 30.1
FOXP3 PE-Dazzle 594
CD25 APC
CD25+FOXP3+ 3.41
CD127 PE-Vio770
CD127- 92.2
Total CD4

Supplementary Figure 6. Flow cytometry strategy for the identification and phenotypic analysis of intestinal CD4$^+$ T cells. (**A**) Single cells were selected based on forward and side scatter parameters, and dead cells were excluded using LIVE/DEAD™ Fixable Blue viability dye. Among live cells, CD45$^+$ leukocytes were gated, followed by selection of CD3$^+$ T cells. CD4$^+$ and CD8$^+$ T cell populations were identified within the CD3$^+$ compartment. CD4$^+$ T cells were subsequently classified into memory subsets based on CD45RA and CCR7 expression as follows: central memory (CM, CD45RA$^-$ CCR7$^+$), effector memory (EM, CD45RA$^-$ CCR7$^-$), and terminal effector memory (EMRA, CD45RA$^+$ CCR7$^-$). Within the CD45RA$^+$ CCR7$^+$ population, naïve T cells and T stem cell memory (Tscm) cells were discriminated based on CD95 and CCR7 expression: Tscm (CD45RA$^+$ CCR7$^+$ CD95$^+$) and naïve (CD45RA$^+$ CCR7$^+$ CD95$^-$). Expression of phenotypic markers PD-1, HLA-DR, Ki-67, α4β7, and CD69 was assessed on each subset. Corresponding isotype controls are displayed in the yellow box. (**B**) Gating strategy to identify Th1 (IFN-γ$^+$), Th17 (IL-17$^+$), and Th22 (IL-22$^+$IL-17$^-$) CD4$^+$ T cells in the unstimulated condition (upper panel) and after PMA/ionomycin stimulation (lower panel). (**C**) Gating strategy to identify regulatory T cells (CD25$^+$FOXP3$^+$CD127$^-$) among intestinal CD4$^+$ T cells.

Supplementary figure 7

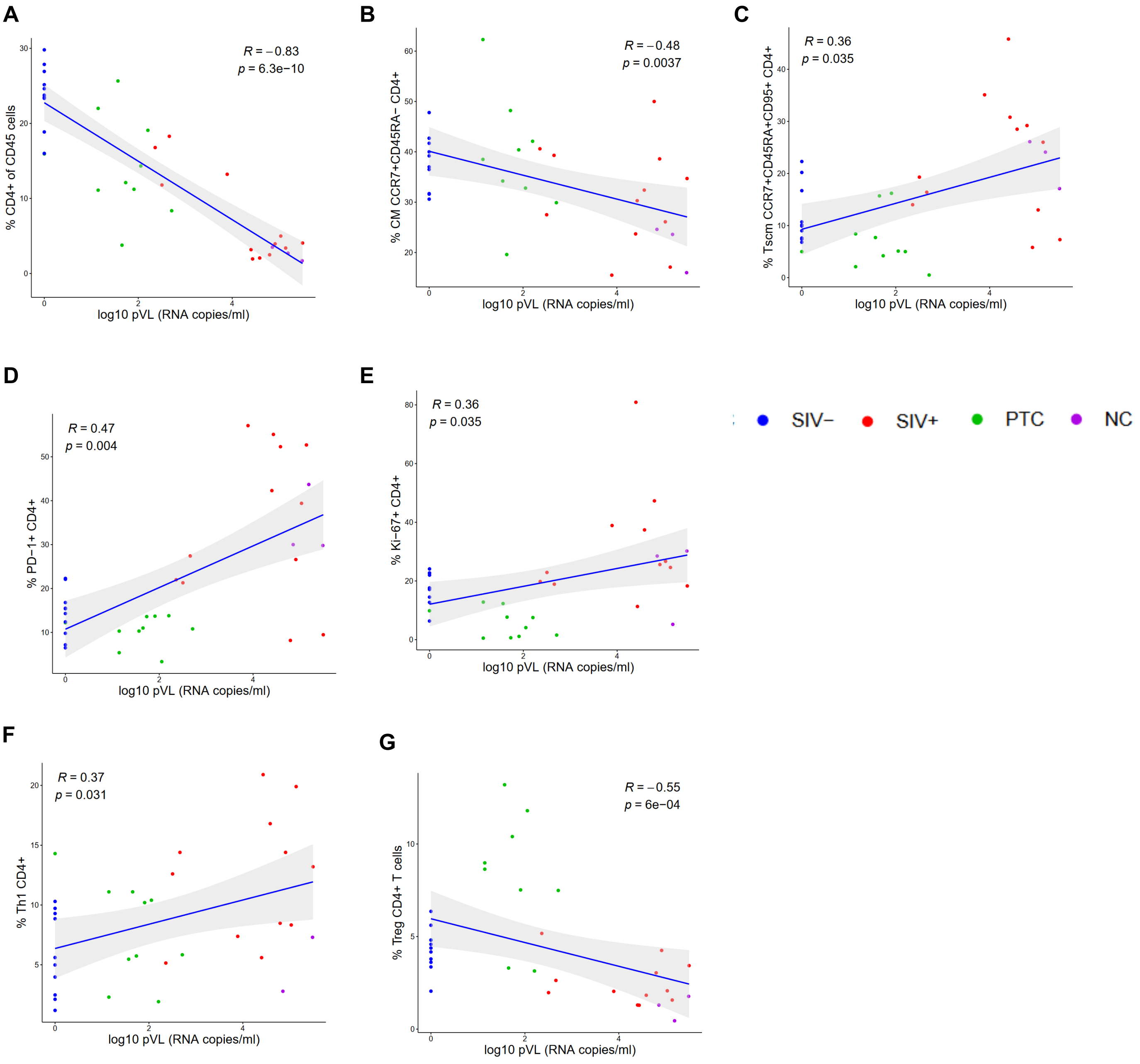

Supplementary Figure 7. Plasma viral load correlates with intestinal $CD4^+$ T cell subset frequencies. (**A–G**) Scatter plots show Spearman correlations between plasma viral load (pVL) and the frequency of $CD4^+$ T cells among total $CD45^+$ cells (A), central memory (CM) $CD4^+$ T cells (B), Tscm $CD4^+$ T cells (C), PD-1^+ $CD4^+$ T cells (D), Ki-67^+ $CD4^+$ T cells (E), Th1 $CD4^+$ T cells (F), and regulatory T cells (Tregs; G) across SIV^-, SIV^+, PTC, and NC animals. Blue lines depict linear regression fits with 95% confidence intervals (gray shading). Spearman correlation coefficients (R) and p-values are indicated in each panel. All Spearman correlation were two-sided. Source data are provided as a Source Data file.

Supplementary figure 8

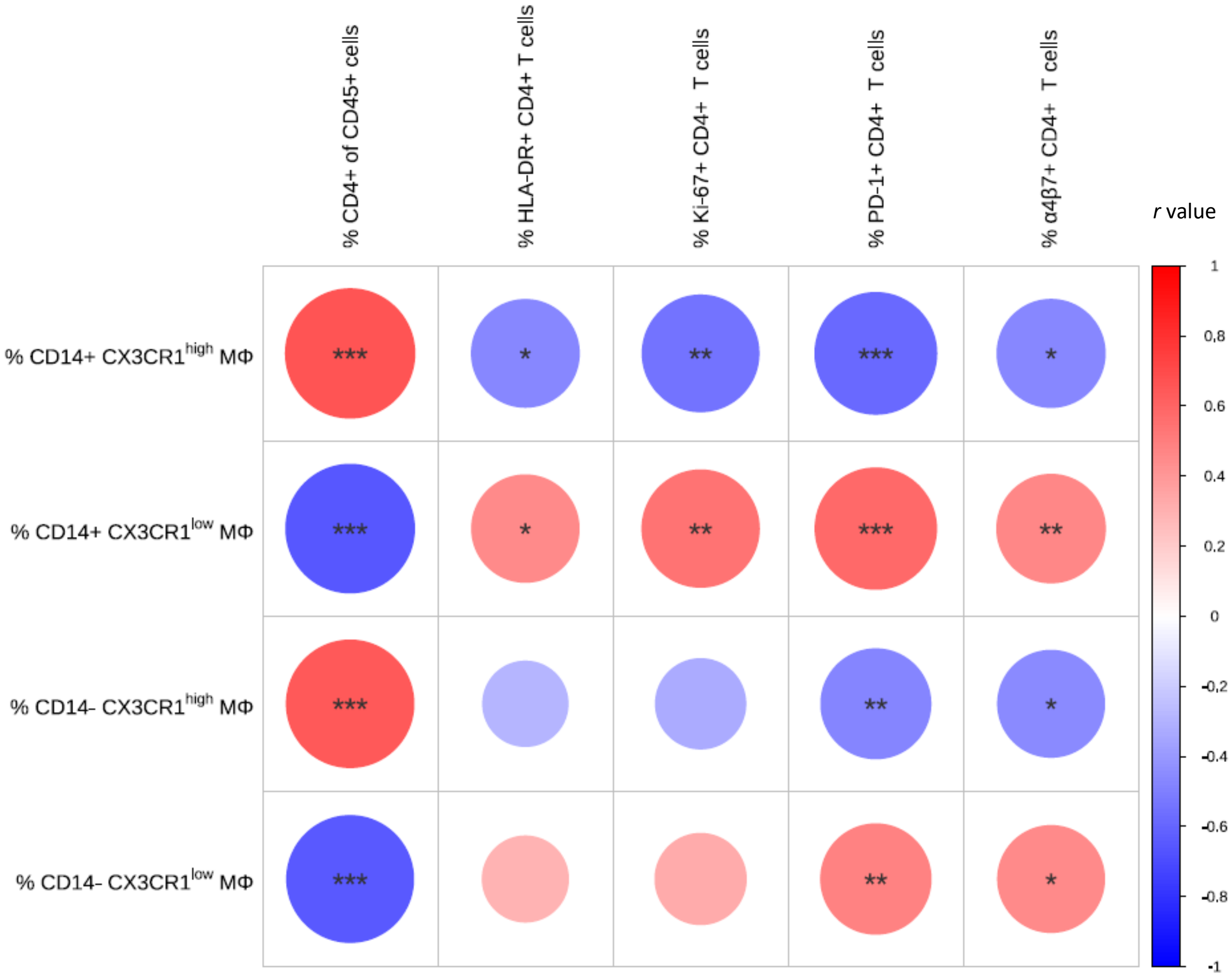

Supplementary Figure 8. Correlation between intestinal CD14⁺ and CD14⁻ macrophage subsets and CD4⁺ T cell activation markers. Heatmap summarizing Spearman correlation coefficients between CD14⁺ and CD14⁻ macrophage subsets, further stratified by CX3CR1^{high} and CX3CR1^{low} expression, and multiple intestinal CD4⁺ T cell parameters: total CD4⁺, HLA-DR⁺, Ki-67⁺, PD-1⁺, and α4β7⁺ CD4⁺ T cells in the sigmoid colon. Spearman correlation coefficients (R) are represented by the color scale (red = positive correlations; blue = negative correlations) and by the size of the circles, which is proportional to the absolute value of R. Statistical significance is indicated by asterisks placed inside each circle (*p < 0.05; **p < 0.01; *p < 0.001). All Spearman correlation were two-sided and no multiple comparison adjustment of the p value of the Spearman correlations was made. Source data are provided as a Source Data file.

Supplementary figure 9

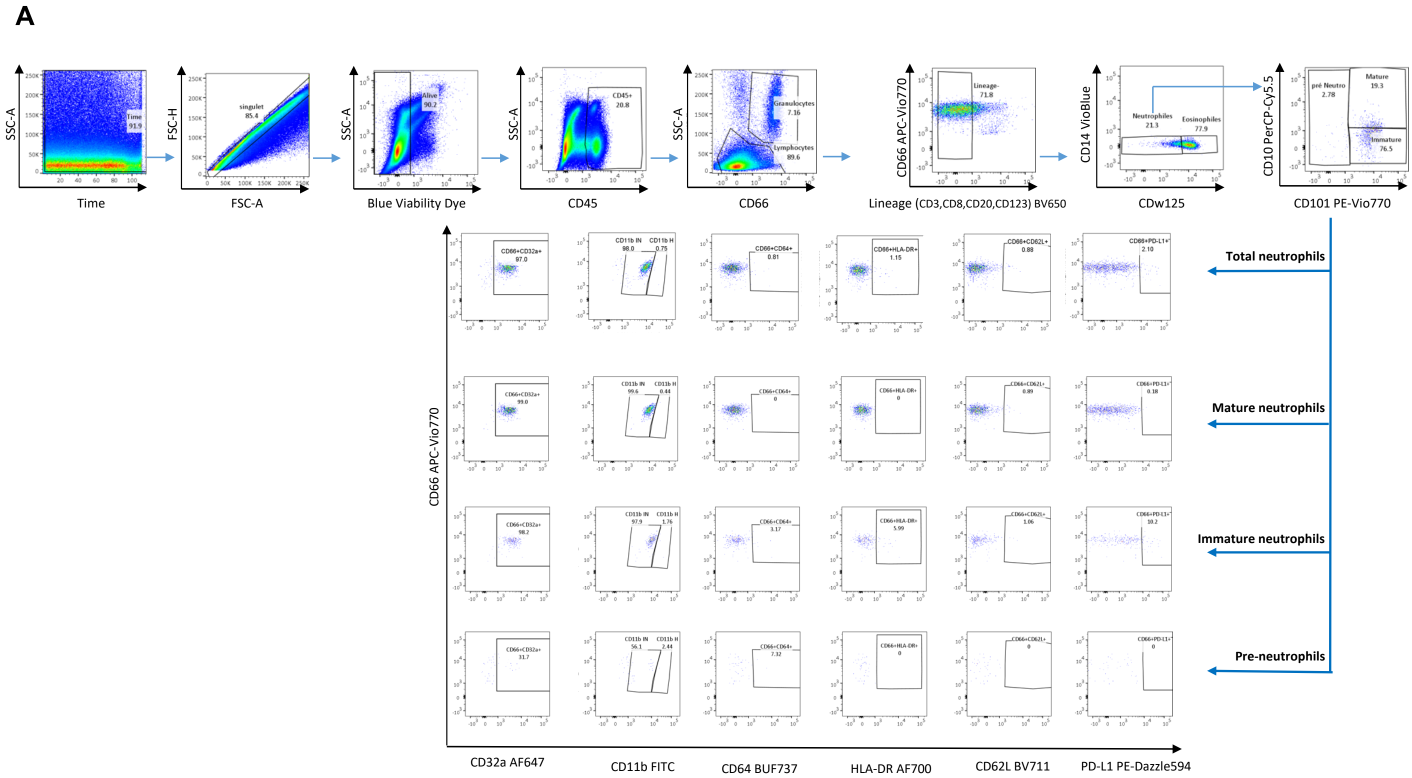

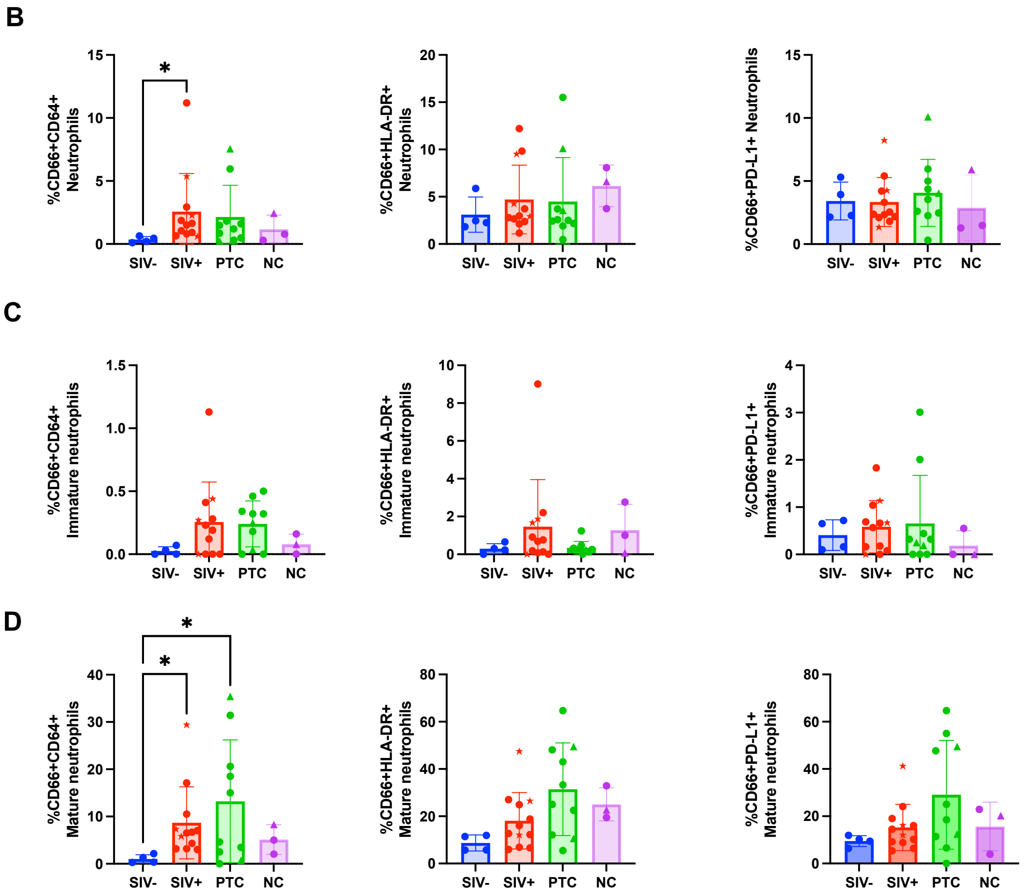

Supplementary Figure 9. Gating strategy and phenotypic characterization of intestinal neutrophils. (**A**) Representative gating strategy used to identify neutrophils in the sigmoid colon. Single cells were first selected based on forward and side scatter parameters, followed by exclusion of dead cells using LIVE/DEAD™ Fixable Blue viability dye. Among live cells, $CD45^+$ leukocytes were gated, and granulocytes were identified as $CD66^+$ cells. Neutrophils were further defined as $CD66^+$ lineage-negative (Lin^-: $CD3^-$ $CD8^-$ $CD20^-$ $CD123^-$ $CD14^-$ $CDw125^-$) cells. Within this population, neutrophil subsets were distinguished based on CD10 and CD101 expression as follows: mature neutrophils ($CD10^+$ $CD101^+$), immature neutrophils ($CD10^-$ $CD101^+$), and preneutrophils ($CD10^-$ $CD101^-$). The expression of CD32a, CD11b, CD64, HLA-DR, CD62L, and PD-L1 was subsequently assessed within each subset. (**B–D**) Expression of CD64 (B), HLA-DR (C), and PD-L1 (D) in total, immature, and mature neutrophil subsets of SIV- (n=4), SIV+ (n=12), PTC (n=10), and NC (n=3) animals. *Kruskal–Wallis with Benjamini–Krieger–Yekutieli (FDR) correction*; $q < 0.05$ (*), $q < 0.01$ (**), $q < 0.001$ (***). Source data are provided as a Source Data file.

Supplementary figure 10

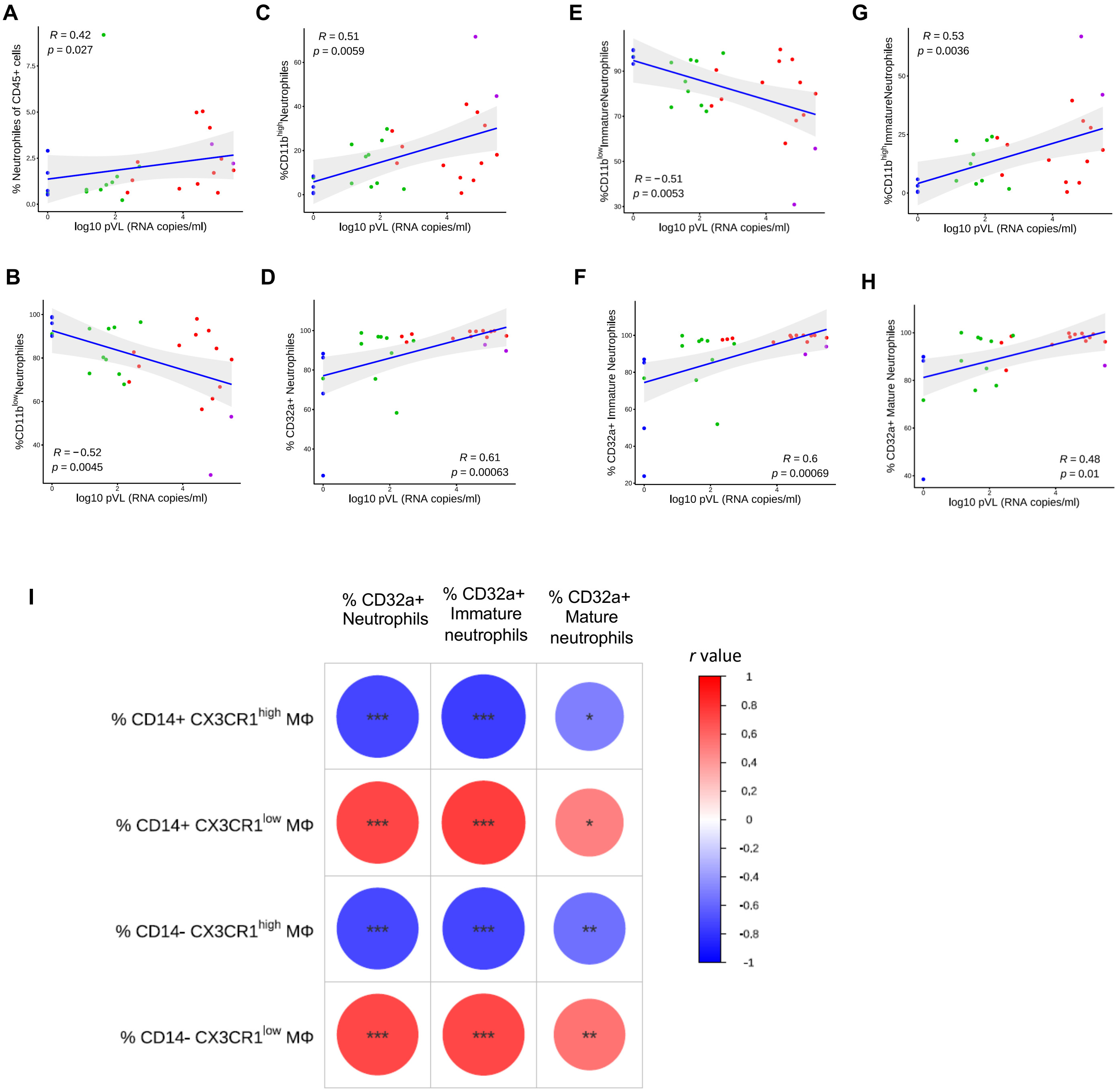

Supplementary Figure 10. Neutrophil activation parameters correlate with plasma viral load and intestinal macrophage phenotypes. (**A–H**) Scatter plots show Spearman correlations between plasma viral load (pVL) and the following sigmoid neutrophil parameters: total neutrophils among CD45$^+$ cells (A), CD11bl^{ow} immature neutrophils (B), CD11b^{high} neutrophils (C), CD32a$^+$ total neutrophils (D), CD11b^{low} total neutrophils (E), CD32a$^+$ immature neutrophils (F), CD11b^{high} immature neutrophils (G), and CD32a$^+$ mature neutrophils (H). Blue lines depict linear regression fits with 95% confidence intervals (gray shading). Spearman correlation coefficients (R) and p-values are indicated in each panel. (**I**) Heatmap showing Spearman correlations between activated neutrophils (CD66$^+$CD32a$^+$) in total, immature, and mature subsets and CX3CR1 expression levels in CD14$^-$ and CD14$^+$ intestinal macrophages. Spearman correlation coefficients (R) are represented by the color scale (red = positive correlations; blue = negative correlations) and by the size of the circles, which is proportional to the absolute value of R. Statistical significance is indicated by asterisks placed inside each circle (*$p < 0.05$; **$p < 0.01$; *$p < 0.001$). All Spearman correlation were two-sided and no multiple comparison adjustment of the p value of the Spearman correlations was made. Source data are provided as a Source Data file.

Supplementary figure 11

Innate cells

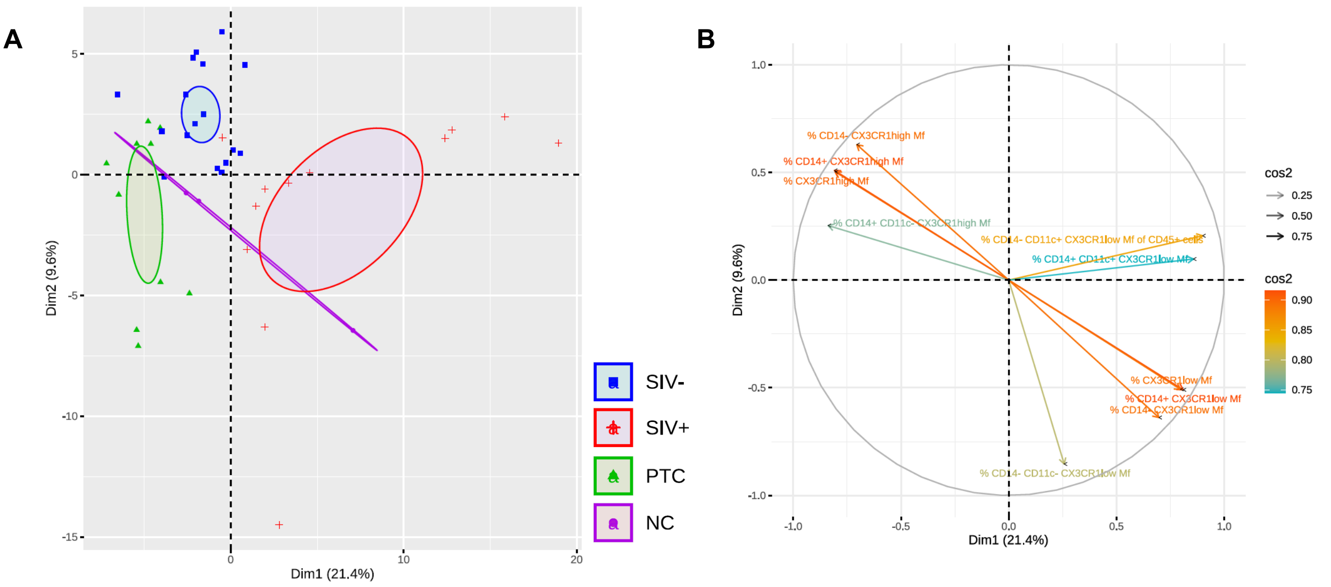

CD4⁺ T cells

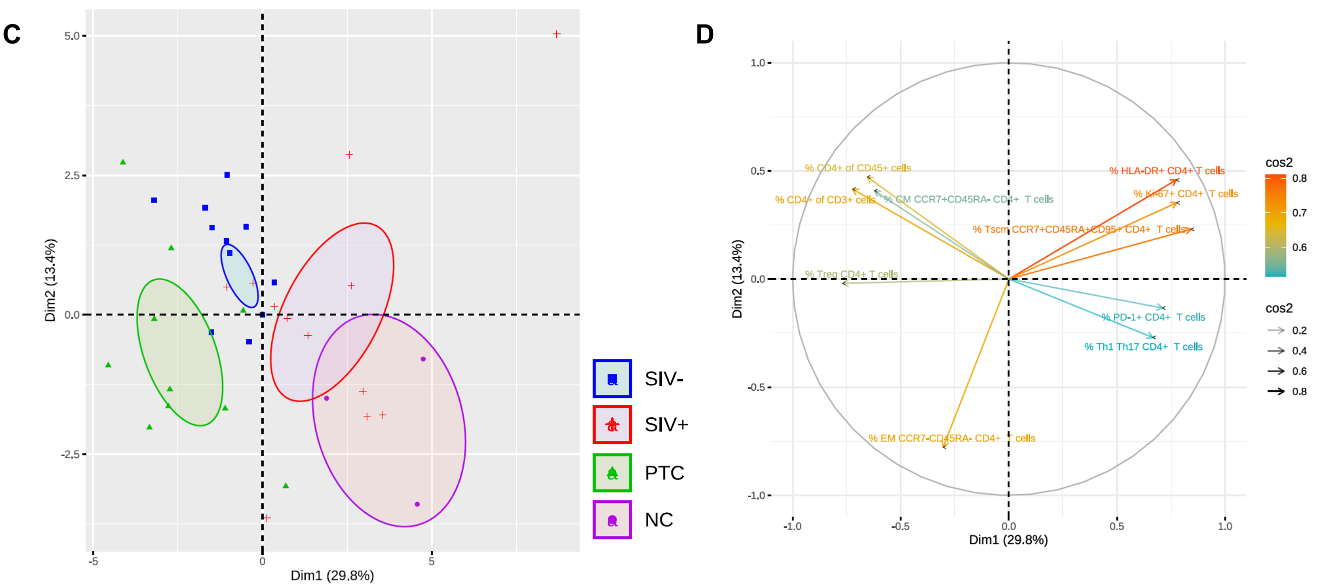

Supplementary Figure 11. Principal component analyses of sigmoid colon immune cells. (**A, C**) Principal component analyses (PCA) of innate immune cell populations, including monocytes, macrophages, dendritic cells, and neutrophils (**A**) and CD4⁺ T cell populations (**C**), in the sigmoid colon of SIV⁻, SIV⁺, PTC, and NC animals. Each dot represents an individual animal. Experimental groups are color-coded: SIV⁻ (blue), SIV⁺ (red), PTC (green), and NC (magenta). PCA was performed in R using normalized frequencies of immune cell populations. **(B, D)** Correlation circles plots showing the contribution of individual variables to the two dimensions for innate immune cells (**B**) and CD4⁺ T cells (**D**). Arrows represent the top 10 contributing variables. Arrow color reflects the squared cosine (cos²), indicating the quality of representation of each variable on the PCA axes: red denotes high cos² (strong representation), while blue indicates low cos² (weak representation).

Supplementary figure 12

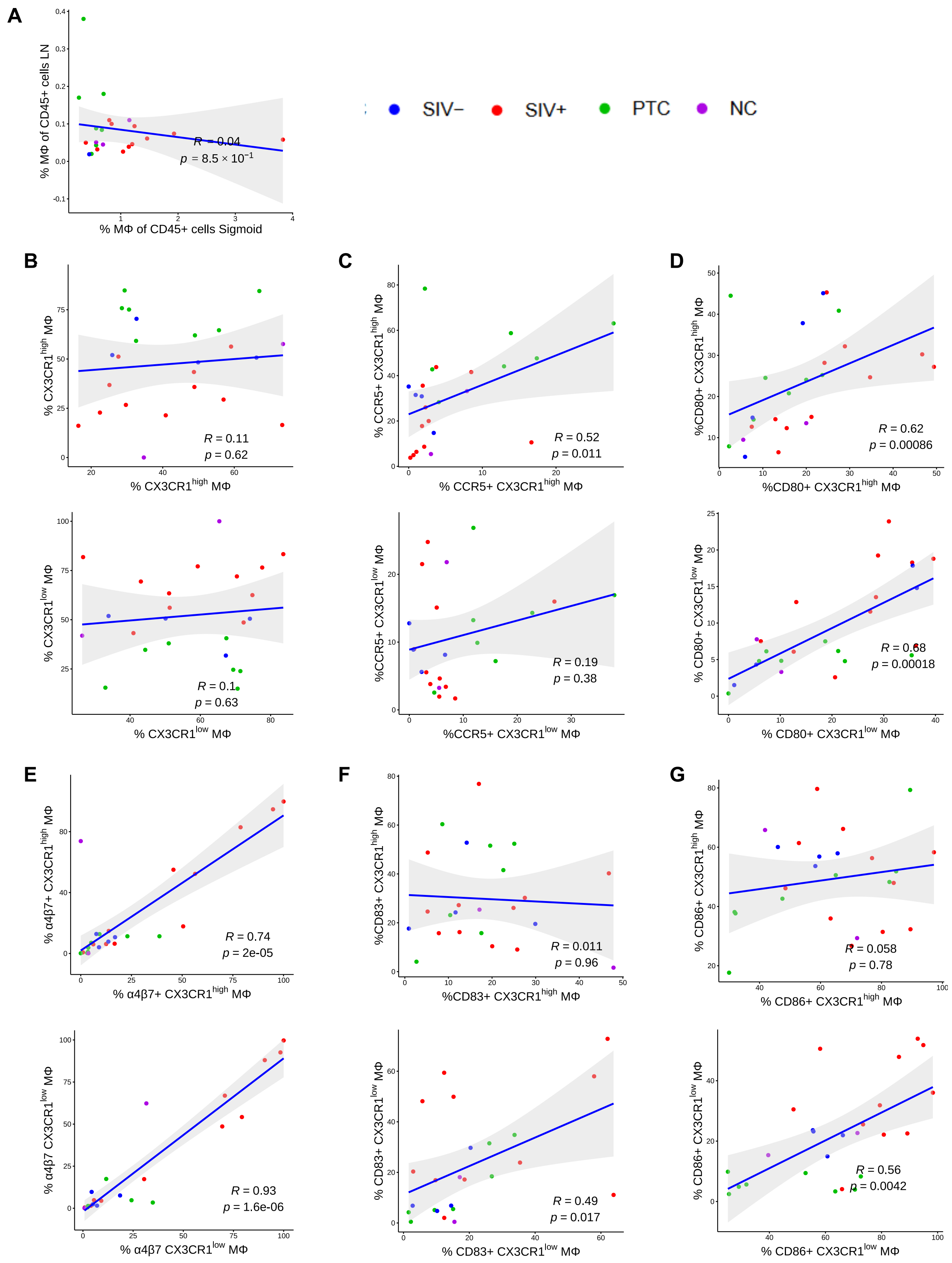

Supplementary Figure 12. Correlations between sigmoid colon and colon-draining lymph node macrophage subsets. Scatter plots show Spearman correlations comparing the frequencies of total Mφ (**A**) and each Mφ subset (**B;** CX3CR1^{high} and CX3CR1^{low};) and their phenotypic markers (**C-G;** CCR5, CD80, $\alpha_4\beta_7$, CD83, CD86) measured in the sigmoid colon (x-axis) versus the corresponding colon-draining lymph nodes (y-axis) from the same animals. Blue lines depict linear regression fits with 95% confidence intervals (gray shading). Spearman correlation coefficients (R) and p-values are indicated in each panel. All Spearman correlation were two-sided. Source data are provided as a Source Data file.
