# Supplementary material for: Maintenance of intestinal CX3CR1+ macrophage homeostasis defines post-treatment control in SIV-infected macaques
Source: Nat Commun. 2026 Feb 24;17:3111. doi: 10.1038/s41467-026-69848-5 (PMC13039934; doi:10.1038/s41467-026-69848-5)
Supplement: Supplementary file 3 — Reporting Summary [file 41467_2026_69848_MOESM3_ESM.pdf]

nature portfolio

Corresponding author(s): Mariangela Cavarelli

Last updated by author(s): Dec 15, 2025

Reporting Summary

Nature Portfolio wishes to improve the reproducibility of the work that we publish. This form provides structure for consistency and transparency in reporting. For further information on Nature Portfolio policies, see our Editorial Policies and the Editorial Policy Checklist.

Statistics

For all statistical analyses, confirm that the following items are present in the figure legend, table legend, main text, or Methods section.

n/a	Confirmed	
☐	☒	The exact sample size (*n*) for each experimental group/condition, given as a discrete number and unit of measurement
☐	☒	A statement on whether measurements were taken from distinct samples or whether the same sample was measured repeatedly
☐	☒	The statistical test(s) used AND whether they are one- or two-sided<br>*Only common tests should be described solely by name; describe more complex techniques in the Methods section.*
☐	☒	A description of all covariates tested
☐	☒	A description of any assumptions or corrections, such as tests of normality and adjustment for multiple comparisons
☐	☒	A full description of the statistical parameters including central tendency (e.g. means) or other basic estimates (e.g. regression coefficient) AND variation (e.g. standard deviation) or associated estimates of uncertainty (e.g. confidence intervals)
☐	☒	For null hypothesis testing, the test statistic (e.g. *F*, *t*, *r*) with confidence intervals, effect sizes, degrees of freedom and *P* value noted<br>*Give P values as exact values whenever suitable.*
☒	☐	For Bayesian analysis, information on the choice of priors and Markov chain Monte Carlo settings
☒	☐	For hierarchical and complex designs, identification of the appropriate level for tests and full reporting of outcomes
☐	☒	Estimates of effect sizes (e.g. Cohen's *d*, Pearson's *r*), indicating how they were calculated

Our web collection on statistics for biologists contains articles on many of the points above.

Software and code

Policy information about availability of computer code

Data collection	Flow cytometry data was collected with FlowJo software version 9.8.3 (Tree Star Inc.).Data storage was ensured by by the BaTLab laboratory management system of of IDMIT and data base interrogation and data visualization were performed using Tableau version 2021.3 (Tableau Software).
Data analysis	Graphs and statistical analyses were performed using Prism version 10.1.2 (GraphPad Software) or R version 4.3.3 (http://www.R-project.org).

For manuscripts utilizing custom algorithms or software that are central to the research but not yet described in published literature, software must be made available to editors and reviewers. We strongly encourage code deposition in a community repository (e.g. GitHub). See the Nature Portfolio guidelines for submitting code & software for further information.

Data

Policy information about availability of data

All manuscripts must include a data availability statement. This statement should provide the following information, where applicable:

- Accession codes, unique identifiers, or web links for publicly available datasets
- A description of any restrictions on data availability
- For clinical datasets or third party data, please ensure that the statement adheres to our policy

The raw data for graphs are available in the Source Data file. Source data are provided with this paper. Additional data are available from the corresponding author Mariangela Cavarelli (mariangela.cavarelli@cea.fr), upon reasonable request.

Research involving human participants, their data, or biological material

Policy information about studies with human participants or human data. See also policy information about sex, gender (identity/presentation), and sexual orientation and race, ethnicity and racism.

Reporting on sex and gender	NA
Reporting on race, ethnicity, or other socially relevant groupings	NA
Population characteristics	NA
Recruitment	NA
Ethics oversight	NA

Note that full information on the approval of the study protocol must also be provided in the manuscript.

Field-specific reporting

Please select the one below that is the best fit for your research. If you are not sure, read the appropriate sections before making your selection.

[x] Life sciences [ ] Behavioural & social sciences [ ] Ecological, evolutionary & environmental sciences

For a reference copy of the document with all sections, see nature.com/documents/nr-reporting-summary-flat.pdf

Life sciences study design

All studies must disclose on these points even when the disclosure is negative.

Sample size	The sample size determined in the pVISCONTI study followed ethical recommendations and good practices for the use of non-human primates in biomedical research. Sample size were defined in order to assume a statistical analyses with a level of significance of 5% (! = 0.05), a target power of 80% (1 - " = 0.8) and an effect size of 1.6 with the objective to use non-parametric tests (Kruskal-Wallis, Wilcoxon rank and Mann Whitney tests) for group comparisons, with Benjamini, Krieger, and Yekutieli FDR approach) to adjust p-values when multiple comparisons are performed.
Data exclusions	Animals included in this study constitute a subset of the original pVISCONTI cohort descibed in Passaes et al. nature Communications 2024
Replication	The pVISCONTI study was performed in two independent experimental phases, each one including 6 W4- and 6 W24-treated macaques that were infected, treated, and monitored in parallel with reproducible results.
Randomization	Animals were allocated to each group to closely match in terms of age, weight, and genotype.<br>Sample collection and analyses were performed in random order
Blinding	The investigators were not blinded as per study design: sampling calendar was adapted to the delay to treatment initiation/treatment interruption for the different groups

Reporting for specific materials, systems and methods

We require information from authors about some types of materials, experimental systems and methods used in many studies. Here, indicate whether each material, system or method listed is relevant to your study. If you are not sure if a list item applies to your research, read the appropriate section before selecting a response.

Materials & experimental systems

n/a	Involved in the study
	[x] Antibodies
[x]	Eukaryotic cell lines
[x]	Palaeontology and archaeology
	[x] Animals and other organisms
[x]	Clinical data
[x]	Dual use research of concern
[x]	Plants

Methods

n/a	Involved in the study
[x]	ChIP-seq
	[x] Flow cytometry
[x]	MRI-based neuroimaging

Antibodies

Antibodies used

The following antibodies were used:a fixable viability stain (Blue, UV excitation; Invitrogen, L23105, 0.5µL); CD45 (PerCP, 2.5µL, clone D058-1283, BD Pharmingen 558411; VioGreen, 2µL, or PerCP-Vio700, 1.5µL, clone REA1023, Miltenyi Biotec 130-117-193 / 130-117-197); CD3 (V500, 3µL, BV650, 2µL, or BUV395, 2µL, clone SP34-2, BD Horizon 560770 / 563916 / 564117); CD4 (BV421, 2µL, clone L200, BD Horizon 562842; VioBright 515, 2µL, clone REA623, Miltenyi Biotec 130-114-535); CD8 (BV650, 2µL, or Alexa Fluor 700, 2µL, clone RPA-T8, BD Horizon 563821 or SONY 2105140); CD14 (Alexa Fluor 700, 1µL, clone M5E2, BD Pharmingen 557923; VioBlue, 2µL, clone REA599, Miltenyi Biotec 130-110-524); CD16 (PE-CF594, 2µL, or BUV395, 2µL, clone 3G8, BD Horizon 562293 / 563785); CD20 (BV650,2µL, or BV711, 3µL, clone 2H7, BD Horizon 563780 / 563126); HLA-DR (APC-Cy7, 2.5µL, clone G46-6, BD Pharmingen 561358; AF700, 1µL, clone L234, BioLegend 307626; APC-Vio770, 2µL, clone REA805, Miltenyi Biotec 130-111-792); CD11c (BV421, 2µL, clone 3.9, SONY 2108140; APC, 5µL, clone S-HCL-3, BD Pharmingen 333144); CD123 (PE-Cy7 2.5µL, or BV650, 2µL, clone 7G3, BD Pharmingen 560826 or BD Horizon 563405); CD64 (V450, 3µL, or BUV737, clone 10.1, BD Horizon 561202 / 564425; VioGreen, 2µL, clone REA978, Miltenyi Biotec 130-116-203); CD103 (PE, 3µL, clone B-Ly7, eBioscience 12-1038); CX3CR1 (FITC, 3µL, clone 2A9-1, BioLegend 341605); CD40 (PE-Dazzle 594, 2µL, clone 5C3, BioLegend 334342); CD83 (APC, 2µL, clone REA714, Miltenyi Biotec 130-110-504); CD86 (APC, 2µL, clone REA968, Miltenyi Biotec 130-116-161); CD80 (BV786, 2µL, clone L307.4, BD Horizon 564159); CD69 (PE-Vio615, 2µL, clone REA824, Miltenyi Biotec 130-112-617); CCR5 (BV786, 2µL, clones G46-17 or 3A9, BD Horizon 565001); CCR9 (PE-Dazzle 594, 2µL, clone L053E8, BioLegend 358918); α4β7 (Alexa Fluor 647, 2µL, clone Hu117, R&D Systems FAB10078R); CD62L (BV786, 2µL, or BV711, 2µL, clone SK11, BD Horizon 565311 / 565040); CD11b (FITC, 2µL, clone REA713, Miltenyi Biotec 130-110-552); CD10 (PerCP-Cy5.5, 5µL, clone HI10a, BioLegend 312216); CD125 (PE, 2.5µL, clone REA705, Miltenyi Biotec 130-110-544); PD-L1 (PE-Dazzle 594, 3µL, clone 29E.2A3, BioLegend 329732); CD101 (PE-Vio770, 2µL, clone REA954, Miltenyi Biotec 130-115-832); CD32a (AF647, 1µL, clone IV.3, Stemcell 60012); CD66abce (APC-Vio770, 2µL, clone TET2, Miltenyi Biotec 130-119-847); CD28 (BV650, 2µL, clone CD28.2, BioLegend 302946); CD95 (BV510, 2µL, clone DX2, SONY 2128200); CCR7 (PE, 1.5µL, clone REA546, Miltenyi Biotec 130-119-583); CD45RA (VioBlue, 2µL, clone T6D11, Miltenyi Biotec 130-113-360); CD25 (APC, 2µL, clone REA945, Miltenyi Biotec 130-115-535); FOXP3 (PE-Dazzle 594, 2µL, clone 206D, BioLegend 320126); CD127 (PE-Vio770, 2µL, clone MB15-18C9, Miltenyi Biotec 130-113-412); Ki-67 (PE-Cy7, 2µL, clone B56, BD Pharmingen 561283); PD-1 (PE-Cy7, 2µL, clone EH12.1, BD Pharmingen 561272); IL-22 (APC, 2µL, clone IL22JOP, eBioscience 17-7222-82); IFN-γ (APC-H7, 2µL, clone B27, SONY 3132620); IL-17A (PE-Vio615, 2µL, clone REA1063, Miltenyi Biotec 130-118-247); and IL-4 (PE-Vio770, 2µL, clone REA895, Miltenyi Biotec 130-114-844).

Validation

Manufacturer's Specifications: We reviewed the manufacturer's specifications and technical data sheets for each antibody. This information included details such as clone names, isotypes, recommended dilutions, and references. Understanding these specifications was essential for proper antibody usage.
Positive and Negative Controls: To validate antibody performance, we used positive and negative controls. Positive controls included cells known to express the target antigen, while negative controls were cells lacking the antigen.
Fluorescence Minus One (FMO) Controls: FMO controls were utilized to assess background fluorescence.
Non-Human Primate (NHP) Reagent Controls: NHP reagent controls were used to ensure the compatibility and specificity of the antibodies in the primate model.

Animals and other research organisms

Policy information about studies involving animals; ARRIVE guidelines recommended for reporting animal research, and Sex and Gender in Research

Laboratory animals

Cynomolgus macaques, imported from Mauritius (median age = 4.8 years at inclusion, IQR = 3.9-7.2).

Wild animals

No wild animals were used in the study

Reporting on sex

Male animals were used in the infected groups, whereas mostly females animals were included in the uninfected group. The conclusions, however, are not sex-based.

Field-collected samples

No field collected samples were used in the study

Ethics oversight

CyMs were imported from Mauritius and housed in facilities at the Infectious Disease Models and Innovative Therapies (IDMIT) center (CEA site at Fontenay-aux-Roses, France). All non-human primate studies at IDMIT are conducted in accordance with French National Regulations under the supervision of National Veterinary Inspectors (CEA Permit Number D92-032-02). IDMIT complies with the Standards for Human Care and Use of Laboratory Animals of the Office for Laboratory Animal Welfare under Assurance Number #A5826-01 and F20-00448. All experimental procedures were conducted according to European Directive 2010/63

(Recommendation Number 9). The pVISCONTI study was approved and accredited under the statement A15 035 from the “Comité d'Ethique en Expérimentation Animale du CEA”, registered and authorized under Number 2453-2015102713323361v3 by the French Ministry of Education and Research. CyMs were studied with veterinary guidance, housed in adjoining individual cages allowing social interactions, and maintained under controlled conditions with respect to humidity, temperature, and light. Water was available ad libitum. Animals were monitored and fed with commercial monkey chow, vegetables and fruits once or twice daily by trained personnel. Environmental enrichment was provided in the form of toys, novel foodstuffs, and music under the supervision of IDMIT Animal Welfare Body. Experimental procedures (animal handling, viral inoculations, and samplings) were conducted after sedation with ketamine chlorhydrate (Imalgene 1000®, 10 mg/kg, intravenously (i.v.), Merial). Tissues were collected during follow up and at necropsy. Animals were euthanized after ketamine chlorhydrate sedation followed by a bolus of sodium pentobarbital (Doléthal, 180 mg/kg, i.v., Laboratoire Vetoquinol).

Note that full information on the approval of the study protocol must also be provided in the manuscript.

Plants

Seed stocks

NA

Novel plant genotypes

NA

Authentication

NA

Flow Cytometry

Plots

Confirm that:

- [x] The axis labels state the marker and fluorochrome used (e.g. CD4-FITC).
- [x] The axis scales are clearly visible. Include numbers along axes only for bottom left plot of group (a 'group' is an analysis of identical markers).
- [x] All plots are contour plots with outliers or pseudocolor plots.
- [x] A numerical value for number of cells or percentage (with statistics) is provided.

Methodology

Sample preparation

PBMCs and tissue samples were analyzed by by Flow Cytometry in in the present study. A detailed protocol for sample processing is indicated in in the methods section, as as follows:

Sample collection and processing

Blood samples were collected in BD Vacutainer Plus Plastic K3EDTA tubes (BD Biosciences) for plasma viral load quantification before viral exposure from sedated animals following 5 mg/kg intra-muscular injection of Zoletil 100 (Virbac, Carros, France), during follow-up, and at necropsy. Plasma was isolated by centrifugation for 10 min at 480 × g and cryopreserved at −80 °C.

Peripheral blood mononuclear cells (PBMCS) were isolated from Vacutainer CPT Mononuclear Cell Preparation Tubes with Sodium Heparin according to the manufacturer’s instructions (BD Biosciences), and red blood cells were lysed in ammonium-chloride-potassium (ACK) buffer (0.15 M NH_4Cl, 16 mM $KHCO_3$, 0.1 mM EDTA, pH 7.4).

The sigmoid colon and draining colon lymph nodes were collected at necropsy.

Lymph node single cells were obtained by mechanical dissociation, passing through a 70 µm nylon filter using gentle pressure applied with a sterile syringe plunger. Lamina propria mononuclear cells (LPMCs) were isolated from fresh intestinal tissues immediately after necropsy, as previously described [107]. Sigmoid colons were cut into small pieces and incubated for 20 min at 37°C in HBSS medium without Ca++/Mg++ (Fisher Scientific, Illkirch, France) supplemented with 5 mM EDTA and 1 mM DTT (Sigma-Aldrich, St Quantin Fallavier, France) to eliminate mucus and epithelial cells. After washing in PBS, the tissue was incubated for 1 h at 37°C with agitation in HBSS medium with Ca++/Mg++ (Fisher Scientific, Illkirch, France) containing collagenase type VIII (0.25 mg/ml, Sigma Aldrich, St Quantin Fallavier, France) and DNase (5 U/ml, Roche, Mannheim, Germany). Undigested pieces were submitted to a second digestion for 30 min. Cell suspensions from both lymph nodes and colon were filtered through 70-µm sterile nylon cell strainers (BD Biosciences), washed with complete medium (RPMI supplemented with 10% FCS, 100 U/ml penicillin/streptomycin, 1% glutamine, 1% NEAA, 1% Na-pyruvate, 1% HEPES buffer [1 M]; all from Fisher Scientific, Illkirch, France), stained on the day of isolation for phenotypic characterization and stored at 4 ° C overnight before flow cytometric acquisition the following morning. For functional assays, freshly isolated cells were stimulated overnight with PMA/Ionomycine (PMA at 62ng/ml and Ionomycine at 720ng/ml; all from Fisher Scientific, Illkirch, France) in the presence of Brefeldin A (10µg/ml from Sigma Aldrich, St Quantin Fallavier, France) and stained the next day. No cryopreservation was applied at any step. Overall, cell recovery and viability were comparable across groups. The total number of isolated cells fell within a similar range for all conditions (mean ± SD: SIV- 85 ± 26 × 10^6, SIV+ 66 ± 30 × 10^6, PTC 114 ± 56 × 10^6, and NC 103 ± 52 × 10^6), and viability remained consistently high, varying between approximately 67% and 80% (mean ± SD: SIV^- 80 ± 5%, SIV^+ 72 ± 5%, PTC 71 ± 9%, NC 67 ± 4%).

Instrument	Cells were acquired using a Fortessa X20 flow cytometer (BD Biosciences) and DIVA software.
Software	Data were analyzed using FlowJo 9.8.3 (Tristar, USA)
Cell population abundance	NA

Gating strategy

The general gating strategy used for all flow cytometry analyses in this study is illustrated in Supplementary Figures 2 and 6. Briefly, events were first gated on singlets based on FSC-A versus FSC-H, followed by exclusion of debris and selection of cells based on morphology using FSC-A versus SSC-A. Dead cells were excluded using a fixable viability dye, and subsequent analyses were performed on live CD45⁺ leukocytes.

T-cell analyses

For T-cell analyses (Supplementary Figure 6), live CD45⁺ cells were gated on CD3⁺ T lymphocytes. CD4⁺ and CD8⁺ T-cell subsets were identified based on CD4 and CD8 expression. CD4⁺ T cells were further classified into differentiation subsets according to the combined expression of CD45RA, CCR7, CD27, and CD95. Naïve T cells were defined as CD45RA⁺CCR7⁺CD27⁺CD95⁻, central memory (CM) cells as CD45RA⁻CCR7⁺CD27⁺, transitional memory (TM) cells as CD45RA⁻CCR7⁻CD27⁺, effector memory (EM) cells as CD45RA⁻CCR7⁻CD27⁻, and terminally differentiated effector cells (EMRA) as CD45RA⁺CCR7⁻CD27⁻. Stem cell memory T cells (Tscm) were identified as CD45RA⁺CCR7⁺CD27⁺CD95⁺.

Phenotypic and functional analyses of CD4⁺ T cells included the assessment of activation markers (HLA-DR and CD38), proliferation (Ki-67), tissue residency and activation markers (CD69 and α4β7), and immune checkpoint expression (PD-1). Intracellular cytokine production was evaluated after stimulation, allowing identification of Th1 (IFN-γ⁺), Th17 (IL-17A⁺), Th22 (IL-22⁺), and Th2 (IL-4⁺) subsets, as shown in Supplementary Figure 6B. Regulatory T cells were identified within the CD4⁺ T-cell compartment as CD25⁺FOXP3⁺CD127⁻ cells (Supplementary Figure 6C).

Negative and positive populations were defined using fluorescence minus one (FMO) controls and appropriate isotype controls. For intracellular cytokine analyses, non-stimulated samples served as negative controls, while PMA/ionomycin- or mitogen-stimulated samples were used as positive controls.

Myeloid cell analyses

For myeloid cell analyses (Supplementary Figure 2), live CD45⁺ leukocytes were first gated to exclude lymphocytes using a CD3⁻CD20⁻ gate, thereby removing T, B, and NK cells. Myeloid cells were subsequently identified as HLA-DR⁺CD14⁺ and/or CD11c⁺ cells. Intestinal macrophages were defined as CD11c⁺CD64⁺ cells and further stratified based on CX3CR1 expression into CX3CR1^high and CX3CR1^low subsets. Conventional dendritic cells (cDCs) were identified as CD11c⁺CD64⁻ cells.

The phenotypic characterization of macrophages included the evaluation of surface markers associated with activation, co-stimulation, homing, and maturation, including CD40, CD80, CD83, CD86, CD69, CCR5, CCR9, α4β7, and CD62L. Isotype controls were used to define background staining and confirm gating thresholds for activation and homing markers, as illustrated in the lower panels of Supplementary Figure 2B.

☒ Tick this box to confirm that a figure exemplifying the gating strategy is provided in the Supplementary Information.
